# Bochdalek hernia with Diamond-Blackfan anemia associated with *RPS19* gene mutation: A case report

**DOI:** 10.1097/MD.0000000000017337

**Published:** 2019-09-27

**Authors:** Ye Seul Yoo, Na Hee Lee, Young Bae Choi

**Affiliations:** a Department of Pediatrics, Chungbuk National University Hospital, Cheongju; b Department of Pediatrics, Cha Bundang Medical Center, Cha University, Seongnam, Korea.

**Keywords:** bochdalek hernia, congenital diaphragmatic hernia, Diamond-Blackfan anemia

## Abstract

**Rationale:**

Diamond-Blackfan anemia (DBA) is a rare inherited marrow disorder, characterized by erythrocyte aplasia and is associated with congenital anomalies and a susceptibility to cancer. Although congenital abnormalities have been observed in ∼50% of DBA patients, the occurrence of an associated congenital diaphragmatic hernia (CDH) has rarely been reported.

**Patient concerns:**

A 19-month-old male child was referred to our pediatric hematology-oncology outpatient clinic with anemic appearance. He presented to us with recurrent anemia, short stature, and developmental delay.

**Diagnosis:**

On bone marrow examination, only erythropoietic cells were markedly decreased in number, whereas other cell lines were unaffected. An abdominal computed tomography scan revealed a Bochdalek type of CDH. A genetic analysis revealed heterozygous mutation of *RPS19*; therefore, he was diagnosed as having DBA with CDH.

**Interventions:**

The patient received an initial packed red blood cell transfusion, followed by an administration of oral prednisone.

**Outcomes:**

The patient is maintained on oral prednisone administered at a dose of 0.3 mg/kg every alternate day and has since a hemoglobin level of >9.0 g/dL without further RBC transfusions.

**Lessons:**

We learned that a Bochdalek type of CDH can manifest in a DBA patient with *RPS19* gene mutation. Therefore, patients diagnosed with the latter disorder should also be screened for an early detection of potential CDHs.

## 1 Introduction

Diamond-Blackfan anemia (DBA) is a rare and inherited marrow disorder, characterized by erythrocyte aplasia, congenital anomalies, and a predisposition to cancer.^[1]^ DBA was first described in 1938 by Louis Diamond and Kenneth Blackfan, and the first causative mutation was identified in 1999, involving the gene *RPS19* which codes for the ribosomal protein S19 located on the chromosome 19q13.2 and os found to be most frequently mutated in these patients.^[2]^ Subsequently, heterozygous mutations resulting in haploinsufficiency in DBA patients have been identified in 19 ribosomal genes, from the small and large ribosomal subunits, RPS and RPL, respectively (*RPS7, RPS10, RPS15A, RPS17, RPS19, RPS24, RPS26, RPS27, RPS28, RPS29, RPL5, RPL11, RPL15, RPL18, RPL26, RPL27, RPL31, RPL35,* and *RPL35A*).^[3]^ Various congenital anomalies including craniofacial abnormalities, thumb deformities, as well as cardiac and renal anomalies have been observed in ∼50% of patients with DBA.^[1]^ However, congenital diaphragmatic hernia (CDH), a structural birth defect owing to a defect in the diaphragm, has been rarely decribed in association with DBA. Here, we report a case of CDH in a patient with Diamond-Blackfan anemia owing to an *RPS19* mutation. This study was approved by the Institutional Review Board of the Chungbuk National University Hospital, and the condition of obtaining an informed consent was waived by the Board because of the retrospective collection of data, which secured the anonymity of the patient.

## 2 Case report

A 19-month-old male child was brought to our pediatric hematology-oncology outpatient clinic for the management of recurrent anemia of unknown cause. There was a history of hydrops fetalis being detected on the patient's fetal ultrasound examination. The patient weight 2.29 kg at birth and had been delivered at 39 + 4 weeks’ gestational age via a vaginal delivery. As a newborn delivery, the patient had cried weakly and had a pale appearance and was consequently diagnosed with respiratory distress syndrome and anemia. He was treated with a surfactant and underwent 6 packed red blood cell (PRBC) transfusions in the neonatal intensive care unit within the first month of his birth. Subsequently, he continued to receive a PRBC transfusion every 3 to 4 weeks for persistent anemia, until he reached 3 months of age. Fortunately, his hemoglobin levels were sustained >9.0 g/dL from the age of 4 months. However, the patient continued to experience wheezing along with repeated episodes of upper respiratory tract infections. At the age of 19 months, there was a recurrence of anemia and he was transferred to the hematology-oncology outpatient clinic. The father informed us regarding the patient's course of admission in a neonatal intensive care unit for the first few months of his life for the management of persistent anemia of an undetermined cause. Other medical history included 2 operations, one each for inguinal hernia and congenital cataract at the ages of 3 and 7 months, respectively. On eliciting the family history, he also disclosed that an older male sibling to the patient had died of apnea at the age of 5 months.

**Figure 1:**
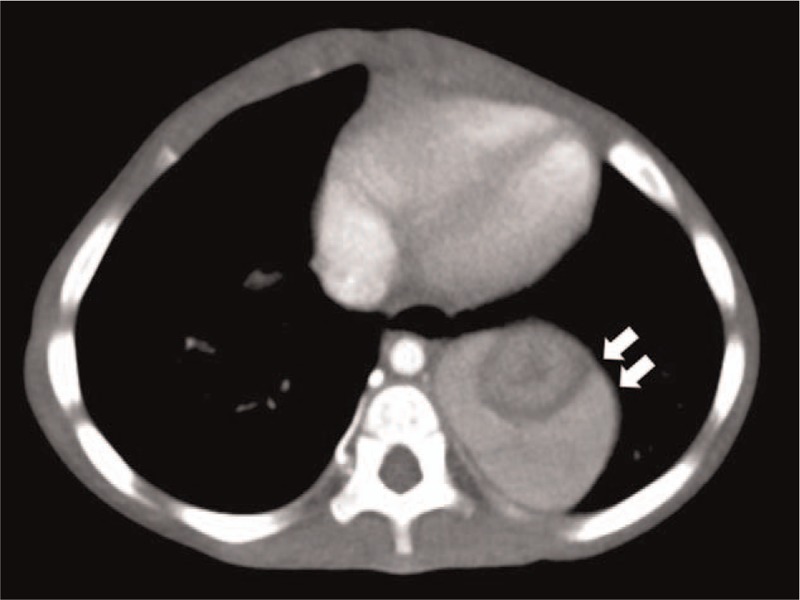
The abdominal computed tomography scan shows a left-sided, posteromedial herniated mass above the diagram (marked with arrow).

On examination, the patient was severely anemic but anicteric. The patient weighted 7.2 kg, had a 75 cm, and his head circumference was 43.5 cm (all parameters <3rd centile for age). His right eye showed strabismus. The systemic examination revealed no abnormalities including hepatosplenomegaly. The developmental milestones for motor skills and language were found to be delayed for age. The patient's hemoglobin (5.4 g/dL; normal range [NR]: 10.5–14.0 g/dL), hematocrit (18.5%; NR: 32–42%), and the reticulocyte count (0.9%; NR: 0.5–1.5%) levels were severely decreased, whereas the mean corpuscular volume was elevated (105.9 fL; NR: 70–90 fL). The total white blood cell and platelet count were normal. On hemoglobin electrophoresis, hemoglobin F level was found to be elevated (12.1%; NR: <1%), along with an increase in the erythrocyte adenosine deaminase level (60.2 IU/L; NR: 8–19 IU/L). The patient's parvovirus B19 serological test (IgM and IgG) results were negative. A computed tomography (CT) scan of the abdomen was performed to rule out other causes of anemia. CT revealed a herniation of stomach and spleen into the thoracic cavity the posteromedial aspect of left hemidiaphragm, and the patient was therefore diagnosed with Bochdalek hernia (Fig. 1). No foci of bleeding and other abnormalities were detected in the CT scan. The bone marrow study revealed a normocellular structure with a very high myeloid-to-erythroid ratio of 18:1 (NR: 1.2:1 to 5:1). The erythropoietic cells were observed to be markedly decreased in number, whereas other cell lines were unaffected. The patient's karyotype was 46XY. An echocardiogram showed an atrial septal defect of 5.58 × 3.77 mm in size. The patient's genetic analysis revealed a heterozygous *RPS19* gene mutation, designated as c.380G>A (p.Gly127Glu), leading to a diagnosis of DBA. The parents refused genetic testing of the other family members. A PRBC transfusion was administered as the initial treatment, following which oral prednisone was administered at a dose of 2 mg/kg/day for 4 weeks and then tapered slowly. The patient was continued on maintenance oral prednisone at a dose of 0.3 mg/kg on alternate days and has since been observed to sustain a hemoglobin level of >9.0 g/dL without receiving further transfusions. As an operative repair of the CDH was not performed at the time, a follow-up of the condition was planned on outpatient basis.

## 3 Discussion

DBA is one of the inherited syndromes responsible for the bone marrow failure and is uniquely characterized by the occurrence of pure erythrocyte aplasia. Although the exact mechanism of DBA has not been fully elucidated, there is evidence that the defective erythropoiesis may be associated with a blockage between the consecutive erythroid burst-forming unit- and the colony-forming unit stages of erythroid development.^[4]^ A ribosomal processing defect in patients with DBA causes this blockade resulting in erythroblastopenia or paucity of the erythropoietic cell line on the bone marrow microscopy. Furthermore, the decreased ribosomal processing activates the p53 apoptotic pathway that induces cell cycle arrest. The outcome of this dysfunction is the DBA phenotype with anemia, short stature, developmental delay, and congenital anomalies including craniofacial abnormalities, thumb deformities, short stature, and cardiac and renal malformation.^[5]^

DBA patients generally present with anemia in the first year of life.^[5]^ Following birth, a normal neonate is exposed to a higher pressure of oxygen as compared to the intrauterine environment, which decreases demand for RBC production, causing a consequent decrease in erythropoietin level. This causes physiologic anemia at 4 to 8 weeks of age, after which, the production of red blood cells gradually returns to normal levels. However, in DBA patients, RBC production is reduced because of a mutational haploinsufficiency of ribosomal synthesis, which results in severe anemia in the first year of life, usually after 8 weeks of age. However, our patient showed hydrops fetalis on the prenatal ultrasound examination and was anemic at birth. Although hydrops fetalis does occur rarely in DBA patients, very few cases have been reported in the literature.^[6–9]^ It is not pinpointed as an exact cause of fetal onset of anemia in DBA patients; further studies are needed to isolate the factors responsible for inciting anemia at the fetal stage.

CDH is classified into 3 types, including posterolateral Bochdalek hernia, an anterior Morgagni hernia, and the hiatus hernia. In DBA patients, craniofacial defects are the most common (representing 50% of congenital malformations associated with the DBA), followed by limb deformities, and cardiac and renal anomalies. The occurrence of CDH in these patients is rare. In previous studies, recurrent deletions of 15q25.2 (on which, the *RPS17* gene is located) have been associated with increased risk of CDH and cognitive deficits, with or without other features of DBA.^[10,11]^ However, there is no recorded case of both Bochdalek hernia and DBA having occurred simultaneously. Although McFarren et al have reported the case of DBA patient with CDH associated with mutations of *RPS19* and *RPS24* genes, the hernia was of a right-sided Morgagni type.^[12]^ Therefore, to the best of our knowledge, this is the reported case of Bochdalek hernia in a DBA patient with *RPS19* mutation.

The treatment of DBA consists of PRBC transfusion, glucocorticoid administration, and hematopoietic stem cell transplantation (HSCT). Although HSCT is the only definitive treatment of DBA, it is not usually preferred for several reasons including the occurrence of regimen-related toxic effects, the risk of graft-versus-host disease, or the possibility of viral reactivation following immunosuppression. Glucocorticoids are the only drugs available for the medical management of DBA with about 80% of the patients responding to the treatment.^[1]^ Initially, a 2 mg/kg/day dose of prednisone for 4 weeks is recommended. If the patient responds favorably, a slow tapering of prednisone is initiated to a level at which, the patient's hemoglobin is maintained at >9.0 g/dL without further PRBC transfusions. Generally, a dosage of 0.5 mg/kg/day or 1 mg/kg/day on alternative days is considered acceptable with respect to long-term glucocorticoid toxicities.^[1]^ However, in patients <1 year of age, glucocorticoids are avoided because of their adverse effects on neurodevelopment and growth.^[13]^ Therefore, in symptomatic DBA patients in this age-group, a PRBC transfusion is recommended every 3 to 5 weeks to maintain the hemoglobin level >9.0 g/dL.^[14]^

In conclusion, we presented our experience in the diagnosis and management of a rare case of Bochdalek type of CDH in a 19-month-old child with DBA because of an *RPS19* mutation. Based on our findings, we suggest that patients with DBA should be screened for CDH.

## Author contributions

**Conceptualization:** Ye Seul Yoo, Young Bae Choi.

**Data curation:** Ye Seul Yoo, Na Hee Lee, Young Bae Choi.

**Writing – original draft:** Ye Seul Yoo, Young Bae Choi.

**Writing – review & editing:** Na Hee Lee, Young Bae Choi.

Young Bae Choi orcid: 0000-0001-7016-8827.
